# Soluble PD-L1 as a Prognostic Factor for Immunotherapy Treatment in Solid Tumors: Systematic Review and Meta-Analysis

**DOI:** 10.3390/ijms232214496

**Published:** 2022-11-21

**Authors:** Fabio Scirocchi, Lidia Strigari, Alessandra Di Filippo, Chiara Napoletano, Angelica Pace, Hassan Rahimi, Andrea Botticelli, Aurelia Rughetti, Marianna Nuti, Ilaria Grazia Zizzari

**Affiliations:** 1 Laboratory of Tumor Immunology and Cell Therapies, Department of Experimental Medicine, “Sapienza” University of Rome, 00161 Rome, Italy; 2 Department of Medical Physics, IRCCS Azienda Ospedaliero-Universitaria di Bologna, 40138 Bologna, Italy; 3 Division of Oncology, Department of Radiological, Oncological and Pathological Science, Policlinico Umberto I, “Sapienza” University of Rome, 00161 Rome, Italy

**Keywords:** programmed death ligand-1 (PD-L1), soluble programmed death ligand-1 (sPD-L1), soluble forms of IC receptors, immunotherapy, prognostic biomarker, survival, solid cancer, meta-analysis

## Abstract

Blocking the Programmed Cell Death Protein 1 (PD-1)/programmed death ligand-1 (PD-L1) axis has demonstrated great efficacy in cancer immunotherapy treatment and remains the central modality of immune targeting. To support the rational and tailored use of these drugs, it is important to identify reliable biomarkers related to survival. The role of the soluble form of the PD-L1 (sPD-L1) as a prognostic biomarker related to survival in solid cancer patients treated with immunotherapy has not yet been consistently evaluated. A systematic literature search of original articles in PubMed, MEDLINE and Scopus was conducted. Studies reporting hazard ratios (HRs) with a 95% confidence interval (CI) or Kaplan–Meier curves or individual patient data for overall survival (OS) or progression-free survival (PFS) associated with baseline levels of sPD-L1 in cancer patients undergoing immunotherapy treatment were considered eligible. Twelve studies involving 1076 patients and different tumor types treated with immunotherapy were included in the analysis. High blood levels of sPD-L1 correlated with poorer OS and PFS in cancer patients treated with immunotherapy (HR = 1.49, 95%CI: 1.15, 1.93, *p* < 0.01, I^2^ = 77% for OS; HR = 1.59, 95%CI: 1.20, 2.12, *p* < 0.01, I^2^ = 82% for PFS). A subgroup analysis highlighted that high levels of sPD-L1 were associated with worse survival in patients affected by NSCLC (HR = 1.81 95%CI: 1.09–3.00, *p* = 0.02, I^2^ = 83% for OS; HR = 2.18, 95%CI: 1.27–3.76, *p* < 0.01, I^2^ = 88% for PFS). An HR > 1 indicated that patients with low levels of sPD-L1 have the highest rates of OS/PFS. In this meta-analysis, we clarified the role of sPD-L1 in different solid cancers treated exclusively with Immune checkpoint inhibitors (ICIs). sPD-L1 could represent a non-invasive biomarker that is easily dosable in the blood of patients. The pooled data from the selected studies showed that a high circulating concentration of sPD-L1 in cancer patients correlates with worse survival, suggesting that it may be a helpful prognostic biomarker for the selection of cancer patients before immunotherapy, thus improving the efficacy of ICIs and avoiding unnecessary treatment.

## 1. Introduction

Immunotherapy has revolutionized cancer treatment in the last decade; indeed, blocking the Programmed Cell Death Protein 1 (PD-1)/programmed death ligand-1 (PD-L1) axis has demonstrated great efficacy in the treatment of cancer and remains the central modality of immune targeting for monotherapy and in combination treatments. Immune-checkpoint inhibitors (ICIs), such as the anti-PD1/PD-L1 blocking antibodies, mainly act on “exhausted” lymphocytes by unleashing their proliferation capacity, restoring function, inducing the release of cytokines and facilitating the activation of an antitumor immune response [1,2]. To date, the expression of programmed death ligand 1 (PD-L1) in tumor tissue remains the most available biomarker to assess patients for treatment with anti-PD1/PD-L1 therapy, although it is not generally considered as an optimal parameter. The analysis of PD-L1 is particularly challenging, mainly due to the dynamic nature of the tumor microenvironment. PD-L1 is a transmembrane protein (also known as CD274 and B7-H1) that is primarily expressed on the cellular surface of antigen-presenting cells and tumor cells. In the tumor microenvironment, PD-L1 recognizes its receptor, PD-1, which is expressed by immune cells such as T and B lymphocytes and myeloid cells and the binding of PD1/PD-L1 induces the proliferation of cancer cells, causing tumor immune escape [3].

Moreover, PD-L1 molecules can also exist in a soluble form (sPD-L1) that is detectable in the blood. Several forms of sPD-L1 have been described: cleaved, secreted splice variants and exosomal [4], and these seem to have several sources. The tumor is not the only source of sPD-L1: several studies have demonstrated that sPD-L1 can also be secreted from antigen-presenting cells such as myeloid dendritic cells in the presence of cytokines and LPS during maturation [5,6]. Moreover, the soluble forms of PD-L1 are biologically active and seems to be able to inhibit T cell functions in various cancers [6,7,8]. The high concentration of sPD-L1 was observed in the serum of cancer patients and may be responsible for immunosuppression or resistance to PD-L1 blockade therapy [9,10]. Therefore, among several factors that are under study as potential biomarkers associated with tumor response to ICI treatment, sPD-L1 has received particular attention due to its identified role as a poor prognostic factor in several cancer types. In fact, increased sPD-L1 levels have been associated with worse prognosis in a wide variety of tumors such as diffuse large B cell lymphoma, T cell lymphoma, multiple myeloma, oral squamous cell carcinoma, melanoma and hepatocellular carcinoma [11].

Previous meta-analyses have suggested that sPD-L1 can predict survival in cancer and that a high concentration of sPD-L1 in blood is associated with worse prognosis [12,13,14]. Nevertheless, the role of sPD-L1 in cancer patients treated with immunotherapy needs to be clarified. Hence, this meta-analysis was conducted by pooling data from selected studies to evaluate the relationship between the value of sPD-L1 in the blood at baseline and the response to immunotherapy, both in terms of progression-free survival (PFS) and overall survival (OS), in cancer patients affected by solid tumors and treated exclusively with ICIs.

## 2. Materials and Methods

### 2.1. Search Strategy and Inclusion Criteria (or Data Identification and Selection)

This meta-analysis was performed following the Preferred Reporting Items for Systematic Reviews and Meta-Analyses (PRISMA) statement. On July 2022, we performed a systematic literature search of PubMed, MEDLINE and Scopus by searching the terms: “Immunotherapy” AND [“cancer” OR “tumor”] AND [“sPDL1” OR “serum” OR “plasma” OR “soluble” OR “blood”] AND [“PDL1” OR “Program death-ligand 1” OR “PD-L1”]. Studies were considered eligible if they reported hazard ratios (HRs) or Kaplan–Meier curves or individual patient data for overall survival (OS) or progression-free survival (PFS). In order to reduce the selection bias, only studies that evaluated sPD-L1 expression by ELISA or Luminex were included. The reference lists of original reports and reviews already published were also analyzed to identify other potential studies. Review articles, case reports, editorials and letters were excluded.

### 2.2. Data Extraction and Statistical Analysis

The estimates of the HRs for PFS and OS were extracted from the studies. When data was presented only as a Kaplan–Meier curve, the WebPlotDigitizer tool (https://automeris.io/WebPlotDigitizer/, accessed on 15 July 2022) was used to estimate data from the figures and extract the proportion of surviving patients at 12 and 24 (if reported) months for patient groups with low or high levels of sPD-L1. The estimated data were also plotted and superimposed on the original Kaplan–Meier curves for final refinement. Individual patients’ data were subsequently pooled.

The natural logarithms of the HRs and standard errors were then calculated based on these measurement points, according to the method described by Tierney et al. [15].

The generic inverse variance method and random-effects models were used to calculate estimates of the average HRs. The publication bias was estimated by inspecting the funnel plots instead of statistical testing, given the small number of included studies [16].

A pooled estimate of HRs was computed according to the inverse variance method. For all analyses, a forest plot was generated to display the results. An HR > 1 indicated that patients with low levels of sPD-L1 had the highest rates of OS/PFS.

In our study, we used a *p* < 0.05 threshold to judge the statistical significance of our findings, which means that the results were statistically significant if the confidence intervals do not include the value of 1 (for HR). We conducted all analyses with the R-package. 

Significant heterogeneity was indicated by a *p*-value < 0.10 in the Cochrane-Q test or an I^2^ statistic value higher than 60%. A random-effects model (REM) was chosen to estimate the pooled estimate of HRs and the impact of baseline sPD-L1 values on the patient outcomes, owing to the expected heterogeneity. The cause of the heterogeneity (if I^2^ > 60%) was investigated using a sub-group analysis (e.g., tumor types). 

The quality of the case-control studies eligible for inclusion was evaluated using the Newcastle-Ottawa Scale (NOS) [17]. Studies with NOS scores of 0–3, 4–6 and 7–9 were considered as low, moderate and high quality, respectively. The publication bias of the included studies was checked using Egger’s test. 

## 3. Results

### 3.1. Studies Selection

**Figure 1:**
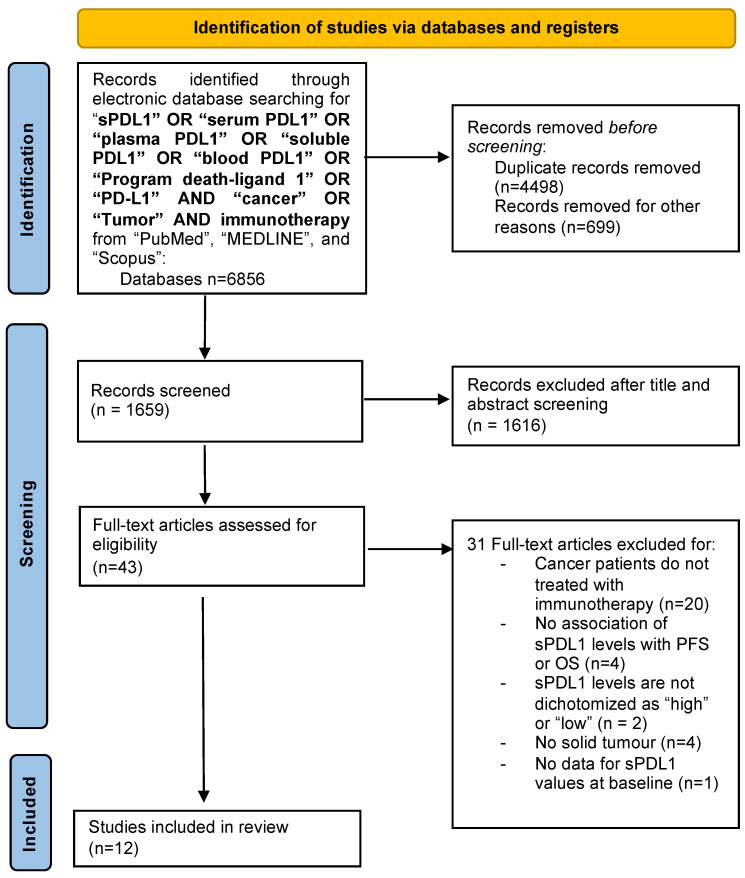
Flow chart of the study selection process.

The study selection process is summarized in Figure 1. In total, 6856 papers were found in the literature search, and after excluding duplicates, abstracts, review articles, letters, mouse studies and chapters of books, 1659 articles were screened. Of these, 1616 manuscripts were further excluded after the title and abstract screening. After full-text screening, 31 full-text articles were excluded for the following reasons: treatment other than immunotherapy (*n* = 20); no investigation between sPD-L1 levels and PFS or OS (*n* = 4); no dichotomy of sPD-L1 levels as “high” or “low” (*n* = 2); no solid tumor (*n* = 4); no data for sPD-L1 values at baseline (*n* = 1). Finally, 12 studies involving several tumors (such as mesothelioma [18], melanoma [19,20], NSCLC [20,21,22,23,24,25,26,27], RCC [20,28]) and a total of 1076 patients met the inclusion criteria and were considered for the review. The details of the studies that fulfilled the inclusion criteria are presented in Table 1. The outcome was sub-categorized according to sPD-L1 values, under or above the threshold reported in the investigated studies (cut-off value).

### 3.2. Studies’ Quality

The quality assessment of the studies was calculated using the Newcastle–Ottawa Quality Assessment Scale (Table 1). Studies with a score ≥6 were considered high-quality. Quality assessment was evaluated by using PFS and OS. Any disagreements were resolved by a discussion among the author group. Twelve studies were evaluated, among them, nine were considered for OS and twelve for PFS. One study [28], which included two cohorts of patients with two different cancers, was considered twice based on the type of cancer analyzed. Four out of 12 studies provided a multivariate HR with a 95% confidence interval based on univariate analysis (although in one study, different values were reported in the table and in the text), of which one was based on multivariate analysis. The remaining studies did not provide HRs but provided Kaplan–Meier figures for OS and PFS, which were used to calculate the HR and 95% confidence interval (Table S2). 

The inspection of the funnel plots showed an asymmetry for both the investigated outcomes that was caused by between-study heterogeneity, indicating a publication bias (Figure S1). In agreement, the statistical heterogeneity was high (>60%), mainly due to the small number of studies included and the different tumor types.

### 3.3. High Blood Levels of sPDL-1 Result Associated with Poorer OS and PFS in Cancer Patients Treated with Immunotherapy

**Figure 2:**
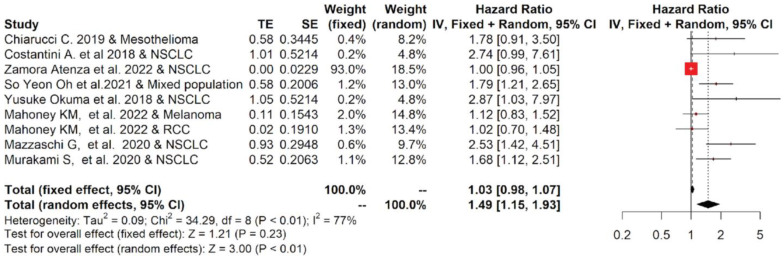
Overall pooled HRs comparing the high- versus the low-PD-L1 values group for OS in patients with solid cancer treated with immunotherapy. The column study indicates the reference (from which we extracted or calculated the HR and 95% CI) and the investigated tumor type [18,20,22,23,24,25,26,27,28].

Nine studies reporting the OS of 880 cases were included in the meta-analysis. Among the studies reporting the OS, the one-year OS average(range) was 62.2 % (55.6–69.7%) and 34.9% (14–55%) for cancer patients with low and high values of sPD-L1, respectively, while the two-year OS average(range) was 38.0% (19.6–54%) and 16.8% (0–30%), respectively (Table S1). Moreover, the OS data were pooled, and the results are shown in Figure 2. The forest plot reported that a high concentration of sPD-L1 in the blood of cancer patients treated with immunotherapy at baseline was associated with reduced OS. The median (range) of the overall pooled HRs comparing the high- versus low-sPD-L1 values group based on the random-effects model was 1.49 (95%CI: 1.15, 1.93, *p* < 0.01, I^2^ = 77%).

**Figure 3:**
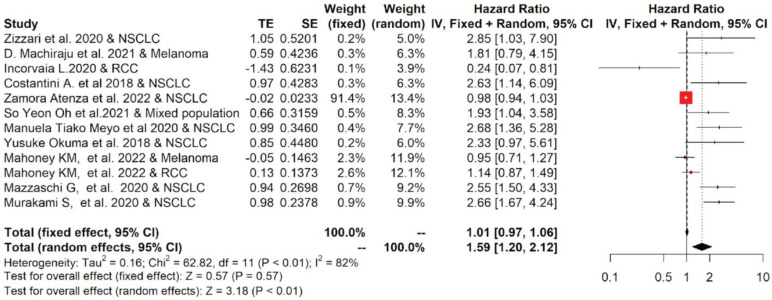
Overall pooled HRs comparing the high- versus the low-PD-L1 values group for PFS in patients with solid cancer treated with immunotherapy. The column study indicates the reference (from which we extracted or calculated the HR and 95% CI) and the investigated tumor type [18,19,20,21,22,23,24,25,26,27,28,29].

Among studies reporting the PFS, the one-year PFS average(range) was 38.5 % (24.8–50%) and 27.5% (0–100%) for cancer patients with low and high values of PD-L1, respectively; while the two-year PFS average(range) was 27.5% (0–100%) and 9.0% (0–21.6%), respectively (Table S1). The forest plots of the meta-analysis conducted on the PFS data are shown in Figure 3. The pooled data of 12 studies showed that high values of sPD-L1 in serum/plasma of cancer patients were also correlated with worse PFS. The median (range) of the overall pooled HRs comparing the high- versus the low-sPD-L1 values group based on the random-effects model was 1.59 (95%CI: 1.20, 2.12, *p* < 0.01, I^2^ = 82%). 

Thus, our analysis indicated that the value of sPD-L1 in the blood can be considered a prognostic factor for OS and PFS in cancer patients with advanced disease treated with immunotherapy.

### 3.4. NSCLC Subgroup Analysis Reveals That High Blood Levels of sPD-L1 Are Correlated with Poor Survival Outcome

**Figure 4:**
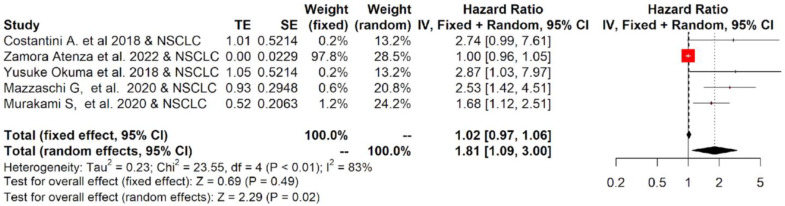
Overall pooled HRs comparing the high- versus the low-PD-L1 values group for OS in NSCLC patients treated with immunotherapy. The column study indicates the reference (from which we extracted or calculated the HR and 95% CI) and the investigated tumor type [22,23,25,26,27].

**Figure 5:**
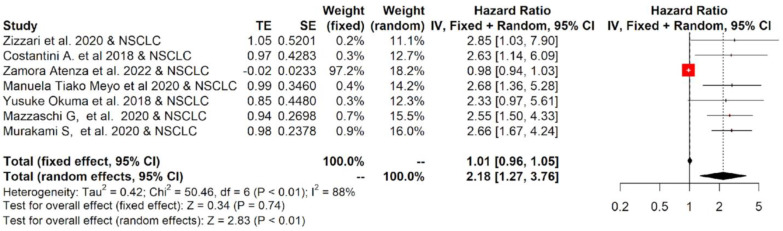
Overall pooled HRs comparing the high- versus the low-PD-L1 values group for PFS in NSCLC patients treated with immunotherapy. The column study indicates the reference (from which we extracted or calculated the HR and 95% CI) and the investigated tumor type [21,22,23,24,25,26,27].

The twelve studies included in the meta-analysis mainly considered three different tumor types treated with immunotherapy: NSCLC, RCC and melanoma. Therefore, a subgroup analysis was performed for each tumor. The subgroup meta-analysis, which pooled data for OS from five studies, including 542 patients affected by NSCLC, indicated that a higher concentration of sPD-L1 in the blood of these patients was significantly associated with worse survival, with an HR = 1.81 (95%CI: 1.09–3.00, *p* = 0.02, I^2^ = 83%; Figure 4). Similar results were obtained for PFS: seven studies that included 616 patients revealed that high levels of sPD-L1 correlated with unfavorable survival in the NSCLC group with an HR = 2.18 (95%CI: 1.27–3.76, *p* < 0.01, I^2^ = 88%, Figure 5). These data were not confirmed for RCC and melanoma patients. In particular, no statistically significant association between sPD-L1 levels and PFS was obtained in the RCC group with an HR = 0.59 (0.13–2.66, *p* = 0.49, I^2^ = 83%, Figure S2) and in the melanoma patients with an HR = 1.16 (95% CI:0.65–2.08, *p* = 0.62, I^2^ = 52%, Figure S3). However, the subgroup meta-analysis conducted on these two tumor types did not have the statistical power to consider whether or not sPDL1 was a prognostic factor. This is due to the limited number of studies included: two for RCC and two for melanoma. Only one study was conducted on mesothelioma patients; therefore, it was not possible to conduct subgroup analyses.

## 4. Discussion

In recent years, the use of ICIs in clinical practice has significantly changed the treatment of advanced cancers. However, the response rates remain widely variable. Intrinsic features of the tumor, the composition of the tumor microenvironment and defects in the host’s innate and adaptive immune system may affect the success of an effective anti-tumor immune response [30]. Hence, it is essential to investigate the possible mechanisms through which the tumor can escape or antagonize the immunotherapeutic efficacy of ICIs. In this context, the identification of novel biomarkers for the selection of patients undergoing immunotherapy treatment represents a crucial issue. Biomarkers that are able to predict the response to immunotherapy in advanced cancers are being studied and some have been clinically validated, such as the expression of PD-L1 on tumor tissue. Nevertheless, the use of these biomarkers has only proved to be useful for some types of cancer. If it is proven that patient responses to anti-PD1/PD-L1 immunotherapy are linearly associated with increased levels of PD-L1 in many types of cancers [31,32,33], then this also demonstrates that the expression of PD-L1 can only partially predict which patients will benefit from therapy. In fact, it was observed that a subset of patients whose tumors lack the expression of PD-L1 benefit from anti-PD-L1 immunotherapy [34]. 

To date, the correlation between the expression of PD-L1 on tumor tissue and the levels of soluble forms of PD-L1 released in the blood of cancer patients is not clearly defined. Several studies have reported no significant association between the membrane-bound PD-L1 and sPD-L1 in primary and advanced NSCLC [22,26]. Conversely, Yang et al. demonstrated a positive correlation between these two parameters in advanced NSCLC patients. Moreover, a study conducted on melanoma patients showed that while PD-L1 was expressed in 67% of tumor biopsies, a soluble form of PD-L1 was detected in the blood of all patients [35]. The advantage is that sPD-L1 may be a more accessible and relevant biomarker compared to the expression of PD-L1 by tumors. Blood tests have the advantage of being minimally invasive, reproducible and they enable monitoring of the immunotherapy treatment. Moreover, sPD-L1 represents a parameter that can act in both the tumor microenvironment and in peripheral blood, inducing local and systemic immunosuppression. In fact, sPD-L1 seems to reduce IFN-γ secretion by T cells and it can participate in systemic anti-tumor immune regulation by targeting T lymphocytes in secondary lymphoid organs [11,35,36].

With this meta-analysis, we wanted to clarify the role of sPD-L1 in correlation with survival related to immunotherapy treatment in cancer patients affected by solid tumors.

Interestingly, it was demonstrated that the levels of sPD-L1 in the blood are higher in cancer patients compared with healthy donors and are correlated to clinical outcomes [6]. In particular, elevated levels of sPD-L1 before ICI therapy, such as anti-CTLA-4 or anti-PD-1 blockade, were associated with an increased likelihood of progressive disease and unfavorable survival in different tumor settings, such as renal cell carcinoma, NSCLC, breast cancer and other solid tumors [6,8,37]. The association between high levels of sPD-L1 and unfavorable survival could be due to a larger tumor burden, increased aberrant splicing activities in tumor cells or an amplified exhausted antitumor immune response. 

Moreover, high expression of sPDL-1 in the peripheral blood of cancer patients could represent a mechanism of resistance that is correlated to the failure of immunotherapy treatment. One hypothesis is that the soluble form of PD-L1 released in the blood of these patients could act as a decoy for the therapeutic anti-PD-L1 antibody, reducing its efficacy [9]. In this context, the use of the anti-PD-1 antibody treatment proved able to overcome the resistance mediated by the sPD-L1 variants. 

**Figure 6:**
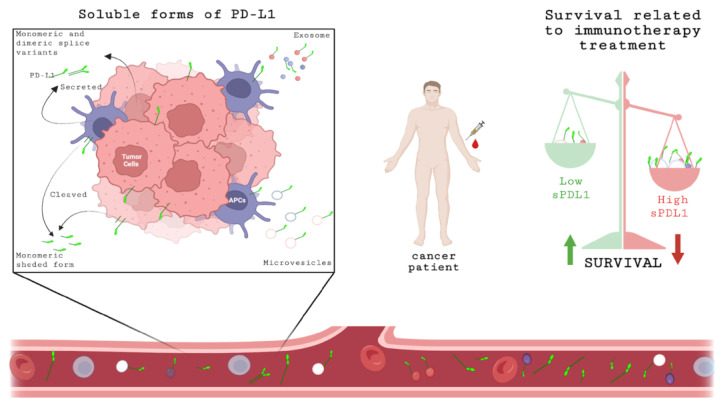
Schematic representation of sPD-L1 expression. Soluble forms of PD-L1 can be released by tumor cells or APCs in different ways: cleaved, secreted monomeric or dimeric splice variants or associated with microvesicles or exosomes. These soluble forms can be detected in serum/plasma. At baseline, before starting immunotherapy treatment, the concentration of sPD-L1 in blood of cancer patient could be a prognostic biomarker related to survival; high levels of sPD-L1 are associated with unfavorable OS and PFS. Created with BioRender.com, accessed on 20 July 2022.

In this meta-analysis, we analyzed, for the first time, the association between sPD-L1 levels in the blood and the prognosis of cancer patients exclusively undergoing immunotherapy treatment in terms of survival, including both OS and PFS. Two selected studies [20,28] also reported a change in the levels of sPD-L1 during immunotherapy treatment. However, the objective of this meta-analysis was to evaluate the role of sPD-L1 prior to the beginning of ICI therapy, so as to understand if this soluble molecule could be used as a valid biomarker to improve the selection of cancer patients who will benefit from treatment. Our results showed that patients with low levels of sPD-L1 at baseline had the highest rates of both OS and PFS, suggesting that sPD-L1 represents a prognostic factor for survival outcome in cancer patients treated with immunotherapy (Figure 6). In fact, higher levels of sPD-L1 at baseline were associated with unfavorable survival. The subgroup analysis revealed that the pretreatment level of sPD-L1 in NSCLC patients receiving ICI treatment is a prognostic factor associated with a poorer clinical outcome. Our results from NSCLC patients were in line with the data obtained by Liao [13]. The number of the selected studies investigating the association between sPD-L1 and OS/PFS in RCC and melanoma patients was limited, therefore the pooled analysis had limited statistical power. One recent meta-analysis demonstrated the prognostic value of sPD-L1 in cancer treated with different therapies [12]. However, in a limited subgroup analysis of three pooled studies, it was observed that high levels of sPD-L1 were associated with poor survival, evaluated as OS, in patients receiving immunotherapy. Therefore, in this meta-analysis, we increased the number of studies selected exclusively for immunotherapy treatment to 12, with a total of 1076 patients, and we obtained statistically significant correlations between the values of circulating sPD-L1 with both OS and PFS. Moreover, to reduce the selection bias, only studies evaluating sPD-L1 concentration by ELISA or Luminex assay were included.

This study has some limitations. Systematic reviewers often encounter incomplete or missing data and the information desired may be difficult to obtain from the study authors. Thus, systematic reviewers may have to estimate data from figures with little or no raw data in the study’s corresponding text or tables. The data extraction enables consideration of estimated data from figures in systematic reviews. The identified program allows accurate evaluations of the actual data from figures with an intraclass coefficient among users of 95%. The heterogeneity of the investigated patient population revealed by I^2^ values was high (>60%); thus, the random-effects meta-analysis was adopted and a subgroup investigation was conducted. Of note, random-effects meta-analyses allow for heterogeneity by assuming that the underlying effects follow a normal distribution. Thus, results must be interpreted considering this assumption (Higgins, Green (editors). *Cochrane Handbook for Systematic Reviews of Interventions* Version 5.1.0 (updated March 2011). The Cochrane Collaboration, 2011. Available from www.handbook.cochrane.org). Moreover, the cut-off values of sPD-L1 in the 12 studies were quite different, contributing to the heterogeneity and limiting the analysis of subgroups for patients with homogenous cut-offs. However, given that sPD-L1 has different sources, such as tumor and immune cells, the heterogeneous levels of this molecule in the blood can reflect several tumor settings, different microenvironments and various immune infiltration profiles. Previous therapies and different molecular signatures from several tumor types could also influence the release of sPDL-1 in the blood. Moreover, although the studies were selected for similar assays, the different kits and biological samples used in the studies could contribute to heterogeneity. In fact, when the same kit (same producer and same catalog number [18,26,27]) with the same biological sample (plasma) was utilized, the cut-off values for sPD-L1 were very similar (Table 1). However, two studies [20,23] that utilized the same kit but different samples (serum or plasma; Table 1) showed different values for sPD-L1. Moreover, the selected studies evaluated sPDL-1 in plasma or serum, analyzed after low-speed centrifugation of whole blood, leaving a cell-free supernatant that contains multiple types of extracellular PD-L1. Consequently, even though this was not the objective of the study, it was not possible to distinguish between and analyze extracellular vesicle-associated, exosomal, shed or secreted forms of PD-L1 molecules, as all forms are present in the blood.

## 5. Conclusions

In conclusion, this meta-analysis shows that sPD-L1 could represent a prognostic biomarker for survival in cancer patients undergoing immunotherapy treatment. In particular, low sPD-L1 was significantly correlated with better OS and PFS. Future ad hoc studies with standard assessment methods could be carried out to define the optimal cut-off value for sPD-L1 and validate its prognostic role in regard to immunotherapy response in cancer. Further studies are also needed on different solid tumor settings to better clarify the role of sPD-L1 in these tumors. Therefore, sPD-L1 could be a non-invasive, reproducible, and easily measurable biomarker in the blood, able to identify and select patients who will benefit from immunotherapy treatment, which has now become the first-line therapy for many cancers.

## Figures and Tables

**Table 1:** Characteristics of the included studies and Quality Assessment (QA) using the Newcastle-Ottawa Scale (NOS).

ID	Authors & Pub Year [Reference]	Treatment	Tumor Type	N. Pts	N. Healthy Donors	Maximum Follow-Pp Months)	sPD-L1 Cut-Off (pg/mL)	End-Point	Study QA Using NOS	Sample Type	Measurement Assay
1	Zizzari et al., 2020 [21]	anti-PD1	NSCLC	22	N/A	40	20	PFS	6	serum	ProcartaPlex (Thermo Fisher, Waltham, MA, USA)
2	D. Machiraju et al., 2021 [19]	anti-CTLA4 (24) anti-PD1 (48) combo (42)	Melanoma	113	N/A	40	133	PFS	6	serum	ELISA (LS Bio, Seattle, WA, USA)
3	Incorvaia L. 2020 [29]	anti-PD1	mccRCC	21	N/A	30	660	PFS	6	plasma	ELISA (homemade)
4	Chiarucci C. 2020 [18]	Anti-PDL1 plus anti-CTLA4	Mesothelioma	40	22	40	70	OS	6	serum	ELISA (R&D System, Minneapolis, MN, USA)
5	Costantini A. et al., 2018 [22]	Anti-PD1	NSCLC	43	N/A	20	33.97	OS/PFS	8	plasma	ELISA (Abcam, Cambridge, UK)
6	Zamora Atenza et al., 2022 [23]	Anti-PDL1 (104) Combo ICI (4)	NSCLC	108	29	60	12.94	OS/PFS	8	plasma	ELISA (Invitrogen, Waltham, MA, USA)
7	So Yeon Oh et al., 2021 [20]	Anti-PD1 (73) Anti-PDL1 (19) Anti-CTLA4 (5) Combo (31)	NSCLC (50) Melanoma (31) SCLC (14) UCC (13) RCC (6) HNSCC (5) Others (9)	128	20	50	11,000	OS/PFS	8	serum	ELISA (Invitrogen)
8	Meyo M. T. et al., 2020 [24]	Anti-PD1	NSCLC	51	36	26.3	156	PFS	8	plasma	ELISA (Cloud-clone Corp, Katy, TX, USA)
9	Okuma Y. al., 2018 [25]	Anti-PD1	NSCLC	39	N/A	15	3357	OS/PFS	8	plasma	ELISA (Cloud-clone Corp)
10	Mahoney KM, et al., 2022 [28]	Anti-PD1	RCC (91) MELANOMA (87)	169	N/A	N/A	1978 (RCC) & 2312 (Melanoma)	OS/PFS	6	serum	ELISA SIMOA assay (Quanterix, Billerica, MA, USA)
11	Mazzaschi G. et al., 2020 [26]	Anti-PD1 (87) Anti-PDL1 (22)	NSCLC	109	N/A	30	113	OS/PFS	8	serum	ELISA (R&D System)
12	Murakami S. 2020 [27]	Anti-PD1	NSCLC	233	N/A	36	90	OS/PFS	7	serum	ELISA (R&D System)

## Data Availability

The data are available on reasonable request from the corresponding author.

## Supplementary Materials

The supporting information can be downloaded at: https://www.mdpi.com/article/10.3390/ijms232214496/s1.
