# Supplementary material for: Soluble PD-L1 as a Prognostic Factor for Immunotherapy Treatment in Solid Tumors: Systematic Review and Meta-Analysis
Source: Int J Mol Sci. 2022 Nov 21;23(22):14496. doi: 10.3390/ijms232214496 (PMC9696773; doi:10.3390/ijms232214496)
Supplement: Supplementary file 1 [file ijms-23-14496-s001.zip › supplementary materials.pptx]

## Slide 1
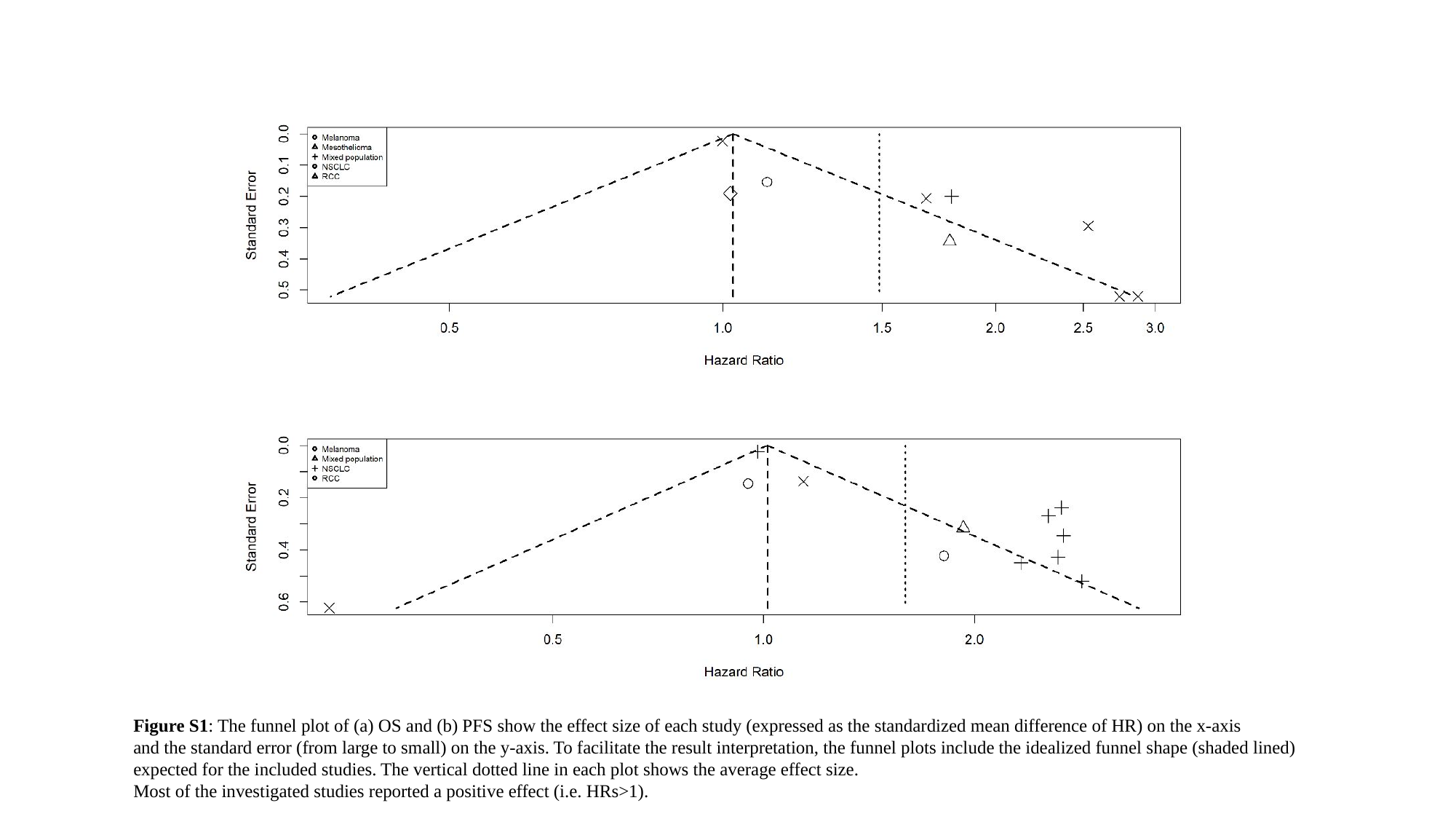

Figure S1: The funnel plot of (a) OS and (b) PFS show the effect size of each study (expressed as the standardized mean difference of HR) on the x-axis
and the standard error (from large to small) on the y-axis. To facilitate the result interpretation, the funnel plots include the idealized funnel shape (shaded lined)
expected for the included studies. The vertical dotted line in each plot shows the average effect size.
Most of the investigated studies reported a positive effect (i.e. HRs>1).

## Slide 2
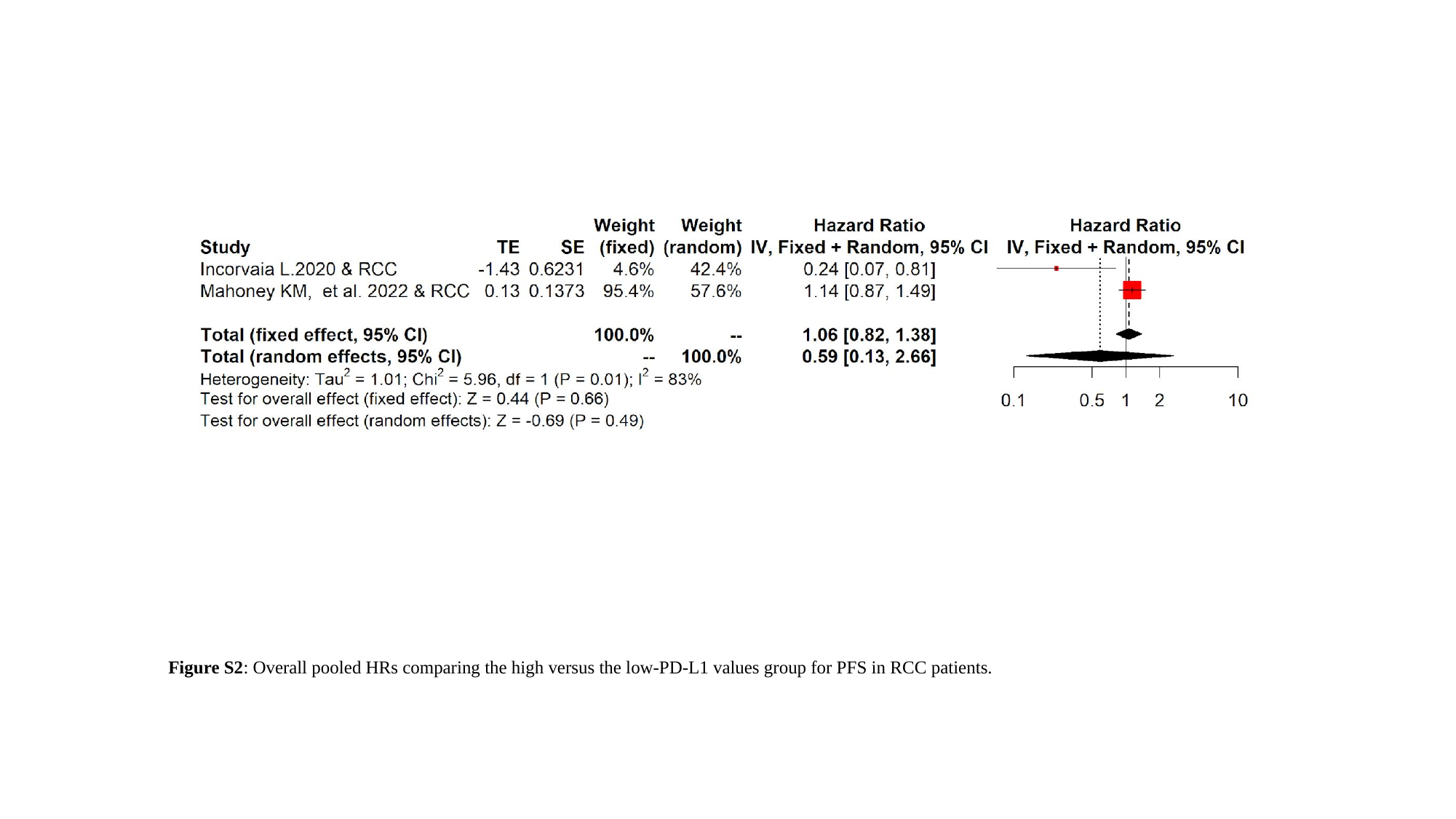

Figure S2: Overall pooled HRs comparing the high versus the low-PD-L1 values group for PFS in RCC patients.

## Slide 3
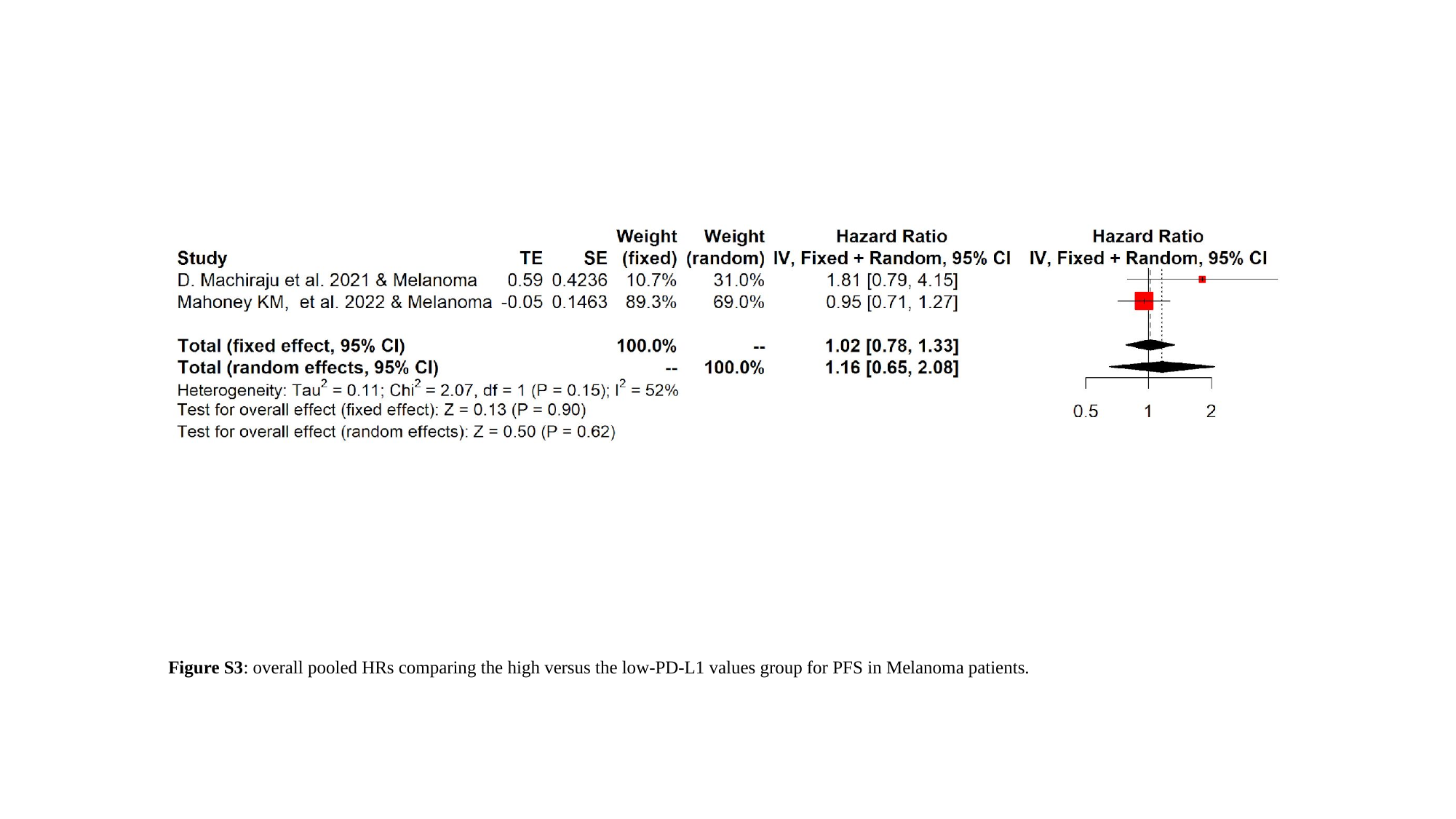

Figure S3: overall pooled HRs comparing the high versus the low-PD-L1 values group for PFS in Melanoma patients.
