# Supplementary material for: Soluble PD-L1 as a Prognostic Factor for Immunotherapy Treatment in Solid Tumors: Systematic Review and Meta-Analysis
Source: Int J Mol Sci. 2022 Nov 21;23(22):14496. doi: 10.3390/ijms232214496 (PMC9696773; doi:10.3390/ijms232214496)
Supplement: Supplementary file 1 [file ijms-23-14496-s001.zip › Table S1+S2.pdf]

Table S1: Information on survival (OS and PFS) data

ID	Authors & Pub Year	N. Pts	1y-OS low	1y-OS high	2y-OS low	2y-OS high	1y-PFS low	1y-PFS high	2y-PFS low	2y-PFS high
1	Zizzari et al. 2020	22	NA	NA	NA	NA	50	18.18182	40	0
2	D. Machiraju et al. 2021	113	NA	NA	NA	NA	NA	NA	NA	NA
3	Incorvaia L.2020	21	NA	NA	NA	NA	25	100	0	11.1
4	Chiarucci C. 2020	40	66.7	47.7	38.1	26	NA	NA	NA	NA
5	Costantini A. et al 2018	43	62.2	41	NA	NA	43.7	21.7	NA	NA
6	Zamora Atenza et al. 2022	119	56	55	41.7	28	37.6	33	31	21.2
7	So Yeon Oh et al.2021	128	59.7	14	19.6	0	24.8	0	12.4	0
8	Manuela Tiako Meyo et al 2020	51	NA	NA	NA	NA	NA	NA	NA	NA
9	Yusuke Okuma et al. 2018	39	69.7	33.4	NA	NA	45.9	20	NA	NA
10	Mahoney KM, et al. 2022	169	NA	NA	NA	NA	NA	NA	NA	NA
11	Murakami S. 2020	233	65.4	34.3	54	30	34.2	27.4	27.6	21.6
12	Mazzaschi G 2020	109	55.6	19	36.8	0	46.8	0	36.4	0
	Mean		62.2	34.9	38.0	16.8	38.5	27.5	24.6	9.0
	Min		55.6	14	19.6	0	24.8	0	0	0
	Max		69.7	55	54	30	50	100	40	21.6

Table S2: input data

ID	Authors & Pub Year	tumor type	hr.mean	hr.lower	hr.upper	end-point	Pts	cut-off PD-L1 (np/mL)
10	Mahoney KM, et al. 2022	Melanoma	1.12	0.83	1.52	OS	78	2312
4	Chiarucci C. 2020	Mesothelioma	1.78	0.92	3.55	OS	40	70
5	Costantini A. et al 2018	NSCLC	2.74	0.86	6.64	OS	33	33.97
6	Zamora Atenza et al. 2022	NSCLC	1	0.96	1.05	OS	118	12.94
9	Yusuke Okuma et al. 2018	NSCLC	2.87	1.11	8.57	OS	39	3357
7	So Yeon Oh et al.2021	Mixed population	1.788	1.207	2.65	OS	17	11000
10	Mahoney KM, et al. 2022	RCC	1.02	0.7	1.48	OS	91	1978
11	Mazzaschi G. et al 2020	NSLCC	2.53	1.42	4.51	OS	109	113
12	Murakami S. 2020	NSLCC	1.677	1.12	2.51	OS	233	90
2	D. Machiraju et al. 2021	Melanoma	1.81	0.88	4.63	PFS	113	133
10	Mahoney KM, et al. 2022	Melanoma	0.95	0.71	1.26	PFS	78	2312
1	Zizzari et al. 2020	NSCLC	2.85	1.35	10.37	PFS	22	20
5	Costantini A. et al 2018	NSCLC	2.63	1.03	5.52	PFS	33	33.97
6	Zamora Atenza et al. 2022	NSCLC	0.98	0.94	1.03	PFS	114	12.94
8	Manuela Tiako Meyo et al 2020	NSCLC	2.68	1.36	5.28	PFS	51	156
9	Yusuke Okuma et al. 2018	NSCLC	2.33	1.09	6.31	PFS	39	3357
7	So Yeon Oh et al.2021	Mixed population	1.928	1.038	3.581	PFS	17	11000
3	Incorvaia L.2020	RCC	0.24	0.02	0.23	PFS	21	660
10	Mahoney KM, et al. 2022	RCC	1.14	0.87	1.49	PFS	91	1978
11	Mazzaschi G. et al 2020	NSLCC	2.55	1.5	4.32	PFS	109	113
12	Murakami S. 2020	NSLCC	2.66	1.67	4.25	PFS	233	90
